# Confined cell migration: Insights from *in vitro* and *in silico* methodologies

**DOI:** 10.1063/5.0297695

**Published:** 2026-11-03

**Authors:** Wyatt Quinones, Jackson Huse, Dylan A. Goodin, Zoe Lung, Landon Teer, Hermann B. Frieboes, Joseph Chen

**Affiliations:** 1 Department of Bioengineering, University of Louisville, Louisville, Kentucky 40292, USA; 2 Department of Medicine, University of Louisville, Louisville, Kentucky 40202, USA; 3 UofL Health – Brown Cancer Center, University of Louisville, Louisville, Kentucky 40202, USA; 4 Center for Predictive Medicine, University of Louisville, Louisville, Kentucky 40222, USA

## Abstract

Cell migration is an essential biological process involved in development, wound healing, immune response, and cancer metastasis. Recent advances in imaging techniques *in vivo* have identified confined cell migration as a critical type of locomotion through tissues and vascular tracks. In particular, confined cell migration is crucial in cancer invasion and metastasis, as tumor infiltration necessitates cell migration through confined pores in tissue (1–20 *μ*m) and vascular components (<5 *μ*m). Although cell migration from a biological perspective has been extensively reviewed, recent developments in bioengineering tools such as three-dimensional *in vitro* models and computational modeling have opened new possibilities to study confined migration in normal development and in disease. This review focuses on *in vitro* bioengineered platforms and mathematical models developed to study the mechanisms of confined migration. We anticipate that further development of these bioengineered platform technologies, coupled with advances in computational modeling, including multi-scale models and machine learning, will be pivotal to facilitating mechanistic testing and evaluation of therapeutic strategies effectively targeting wound healing and cancer metastasis.

## INTRODUCTION

Cell migration is an essential biological process that plays important roles in development, immune response, and wound healing and at the same time enables cancer invasion and metastasis.^1^ While much progress has been made in the dissection of molecular and biophysical mechanisms of migration, many of these discoveries were elucidated on flat, two-dimensional (2D) substrates of plastic and glass, which do not necessarily recapitulate the complex three-dimensional (3D) *in vivo* environment. Migration *in vivo* presents distinct physical challenges, which include extracellular matrix (ECM) networks, anatomical features, and interactions with neighboring cells. Cells dynamically employ multiple modes of migration to navigate this complex environment, and although migration strategies differ significantly depending on the 3D context, the foundational mechanisms dictating cell locomotion are conserved and include actomyosin regulation of cell adhesion, protrusion formation, and contractility.^1^

In the *in vivo* environment, most cells experience confinement as they are continuously embedded in a cellular or ECM context. Within this microenvironment, cells dynamically adapt to efficiently migrate by remodeling the ECM via proteolytic degradation or physical deformation, or bypassing ECM remodeling altogether, choosing opportunistic paths present within the matrix through bleb-based propulsion. The successful transit of cells through this environment is called confined migration, and its study is informed by a variety of mechanical constraints observed *in vivo*, including lateral and vertical confinement, micrometer-sized pores, fiber-guided migration paths, and varied adhesive islands. Within these physical barriers and migration paths, cells modulate cytoskeletal mechanisms to adjust adhesion dynamics, contractility, and nuclear deformation.^2^ Migrating cells sense confinement via mechanosensitive proteins and channels and spatially regulate actomyosin contractility to facilitate mesenchymal or amoeboidal modes of migration.^3^ In confined environments amenable to actin-driven protrusions, mesenchymal migration generally occurs where cells activate adhesion-dependent migration, lamellipodia formation, and matrix degradation to remodel the ECM into a more accessible microenvironment.^1^ However, in tight confinement, cells often adopt a more amoeboidal mode of migration, where cells leverage blebbing-based motility, employ new protrusive structures seen in lobopodial migration and pseudopodial migration, and regulate friction-based migration modes through actin cortex retrograde flow.^4–6^ Cells under tight confinement also alter nuclear mechanics and coordinate nuclear and cytoskeletal components to increase nuclear deformability and limit DNA damage.^7–9^ The mechanisms that cells leverage to navigate confined spaces are still an active area of investigation, with studies focused on the dynamic transitions between migration modes as well as the identification of new models of amoeboidal migration, such as bleb-based “worrying” in melanoma cells, where cell blebbing can physically reorganize the migration path in a protease-independent manner.^10,11^ Continued dissection of confined migration mechanisms has tremendous utility in understanding its role in normal development and in disease, including neural crest (NC) cell and primordial germ cell migration, as well as cancer metastasis and immune cell infiltration.^12^

Investigation of confined migration is challenging, and it has become apparent that examination would ideally benefit from the integration of advanced *in vitro* platforms and computational mechanistic modeling.^13^ The ability to recapitulate physiologically relevant biophysical cues is central to confined migration investigations. A suite of sophisticated microfluidic platforms has been developed to impose physical constraints on cell migration that force cells to employ strategies critical for confined migration, enabling precise dissection of these cellular mechanisms. Additionally, physical descriptive models have been generated based on experimental investigations to detail the interactions between intracellular components, intercellular connections, and the external environment.^14–16^ These models have provided important insights on cytoskeletal and nuclear connections at the single-cell level and have also illuminated leader–follower behavior at a multicellular level.^16–19^ The combination of these strategies has spurred the creation of multi-scale computational models that can incorporate cellular properties such as focal adhesion strength, cytoskeletal traction, and nuclear deformation while at the same time simulate the effect of environmental properties such as viscoelasticity, channel size, and wall friction.^20,21^ The continued development of these tools is expected to advance the understanding of the dynamic mechanisms cells employ to navigate varied 3D environments, with the goal to enable a new suite of invasion-focused pharmacological strategies to support regenerative medicine, mitigate cancer cell infiltration, and support anti-tumor immune cell invasion.

This paper reviews the *in vitro* bioengineered platforms and mathematical models designed to dissect the mechanisms of confined migration. This review highlights seminal works and recent investigations within the last 10 years to survey the role of confined migration in development and disease, reviews bioengineered platforms for confined migration investigations, and describes physical and computational modeling strategies to simulate confined migration in various 3D contexts. Finally, this review offers perspectives on emerging technologies such as machine learning in the pursuit of interrogating confined migration mechanisms and offers outlooks on how advances in experimental and computational approaches will yield insights that can be leveraged for therapeutic purposes.

## CONFINED MIGRATION IN DEVELOPMENT, WOUND HEALING, AND DISEASE

Confined migration plays a critical role in many biological processes, including development, wound healing, immune response, and cancer metastasis.^22^ In each context, migrating cells are confronted with physical barriers that force confined migration and are shaped by chemotactic and biophysical cues. Generally, confined migration occurs through the interplay between cell adhesion and contractility, which is largely informed by the external environment.^19,23^ However, it is important to note that although cells share many mechanisms to navigate confinement, cell-type-specific modes of migration exist that separate fibroblasts, cancer cells, and immune cells. In lower confined environments, cells form actin-based protrusions that can adhere to ECM components and exert traction force and secrete matrix degrading enzymes to remodel the matrix and create more permissive paths.^24^ Cells can then employ ROCK-mediated contractility to translocate through confined spaces.^25^ However, in highly confined environments, cells tend to shift toward high-contractility amoeboidal migration through bleb-based mechanisms.^6^ Blebs are spherical protrusions that do not have filamentous actin but rely on colocalization of actin–plasma membrane linkages and the contractile machinery of the cell.^26^ Bleb-based motility drives contractile forces outwards from the cell body from actin cortex retrograde flow to generate locomotion through friction with the environment, and studies have shown that it is the most efficient mode of migration in confined environments of low adhesiveness.^4,27^ In the complex 3D *in vivo* environment, cells leverage a variety of mechanisms to adapt to the physical obstacles present.^19,27^ Primary human fibroblasts have been shown to employ a nuclear piston mode of migration in confined 3D environments, using actomyosin contractility to pull the nucleus forward and generate high hydrostatic pressure at the front of the cell, which results in lobopodial protrusions. These mechanisms are surveyed below, illustrating the central role confined migration plays in development and disease.

In development, neural crest (NC) migration is a critical process for normal development of the peripheral nervous system, craniofacial structures, and cardiovascular tissues [Fig. 1(a)]. Notably, the NC population must travel large distances in the vertebrate embryo, and abnormal NC migration results in many pathologies that are called neurocristopathies. NC migration begins as NC cells delaminate from the neural tube and migrate in distinct collective streams to reach a target area. Studies have identified two major mechanisms that regulate this collective migration: (1) autocrine-based chemotaxis, which attracts neighboring cells, and (2) contact inhibition of locomotion (CIL), based on the repulsion of the cells at the same time. This dynamic push and pull maintains the collective swarming of NC cells.^28–30^ Intriguingly, recent work has indicated that the maintenance of confined migration requires autocrine-based attraction, CIL, and lateral confinement of cells.^31–33^ In cases where one of these variables is removed, NC collective migration is lost, leading to cluster dispersion.^31,33^ The degree of confinement was also noted to influence efficiency of collective migration, with an apparent optimal combination of confinement width and cluster size predicted by computational models and validated *in vivo*.^34^ The regulator of confinement in the NC migration path is largely regulated by ECM components that create impenetrable borders along the migration route. While fibrous ECM components tend to enable a more penetrable matrix, proteoglycans like versican have been identified as contributors to confinement in NC migration models.^34^ Thus, confined migration is a crucial regulator of efficient NC collective migration, and deeper investigations on these mechanisms can provide insights into the emergence of abnormal NC migration.

In wound healing, epithelial cells, fibroblasts, and immune cells all experience confined migration through inflammatory, proliferative, and remodeling stages [Fig. 1(b)].^41,42^ The inflammatory stage is characterized by the influx of neutrophils and resident immune cells, recruited through growth factors, chemo/cytokines, and bacterial byproducts.^43,44^ Confined migration is required for neutrophils in the peripheral blood to squeeze through the endothelial layer and basement membrane to get to the tissue space.^45^ Notably, neutrophils exhibit dynamic modes of migration with integrin-dependent migration in planar or 2.5D environments and an integrin-independent mode of migration upon exposure to tight confined environments in the injury site. In unconfined environments, neutrophils activate branched actin assembly via Arp2/3 and WAVE-dependent actin assembly; however, under confinement, actin branching is dispensable and is replaced by bleb-based protrusions that are regulated by Rac-dependent control. After 48 h following the onset of injury, monocytes of neighboring blood vessels also infiltrate the lesion area and can either differentiate into cells that present antigens to other immune cells or aid neutrophils in phagocytosis.^43^

In the proliferation phase of wound healing, fibroplasia and contraction of the original wound site occur by vascular remodeling and angiogenesis. In this process, pericytes located in the basal membrane exhibit confined migration to infiltrate into the injury site to regulate angiogenesis. Recent reports have indicated that confinement is central to pericyte phenotype and function, as evidenced by the formation of cell protrusions on the scale of tens of micrometers, a distinct behavior of pericytes *in vivo*.^46,47^ The loss of confinement and migration along 2D planar surfaces creates pericytes with polygonal shapes, prominent actin cables, and flat nuclei, which indicate a loss of *in vivo* phenotypes.^47^ Interestingly, the stiffness and architecture of the surrounding matrix not only influence migration but also affect pericyte function. A recent report indicated that pericytes within stiff and more confined environments reduced endothelial adhesion and vascular barrier function and enhanced genes associated with inflammation and remodeling.^48^ Thus, a proper balance and persistence of biophysical cues at the injury site is crucial for proper pericyte function and vascularization.

In the remodeling stage of wound healing, fibroblasts differentiate into myofibroblasts to facilitate ECM deposition and remodeling as well as wound contraction and tissue alignment before transitioning back into a quiescent phenotype.^49,50^ Immune cells are also active in this stage, migrating through the basement epithelium to clean out damaged cells and prevent infection from the outside environment.^51^ Immune cells and fibroblasts must experience confinement to navigate the wound site, often comprised of a variety of ECM components and a disjointed architecture. Fibroblast confined migration is influenced by a combination of chemotactic and haptotactic signals and is driven by non-muscle myosin II contraction.^52^ Several reports identified significant nuclear deformation in fibroblasts at the injury site and have noted its influence on cytoskeletal organization, mechano-signaling, and ECM remodeling through alterations in chromatin organization and gene expression.^53,54^ Fibroblast confined migration has also been linked to the accumulation of reactive oxygen species (ROS), highlighting its role in regulating the inflammatory microenvironment.^55^ Collectively, fibroblast confined migration appears indispensable for a normal healing process, regulating fibroblast activation and function through a combination of mechanical and soluble factors while simultaneously regulating the inflammatory environment. However, elevated or continual exposure to confinement may also dysregulate healing, generating persistent myofibroblasts and enhanced ROS production. Additional investigations into the role of confined migration on fibroblast activation and function are needed, given their crucial role in wound healing.

In cancer, confined migration is a crucial driver of cell invasion and metastasis [Fig. 1(c)].^56^ As cancer cells spread, they encounter obstacles, including low-diameter capillaries, basal membranes, endothelial layers, and the dense ECM in the tumor microenvironment (TME).^57,58^ Many of these features have pores or tracks that are often smaller than the diameter of a cell, forcing cells to undergo confined migration.^58,59^ When navigating the dense microenvironment, cells adopt various mechanisms to invade by remodeling the ECM via matrix degrading enzymes, physically deforming ECM components, and by adopting amoeboidal modes of migration, which enable cells to squeeze through bleb-based processes.^1,13,19^ In all cases, cells undergoing confined migration will experience significant nuclear deformation, and the extent of nuclear deformation represents the rate-limiting step for successful confined migration.^60^ Studies have suggested that in general, pore sizes that are ~10% of the nuclear cross section area of a cell will restrict confined migration.^25^ Nonetheless, cells can modulate nuclear stiffness to enhance nuclear translocation and successful confined migration through alterations in lamin proteins, key components of the nuclear lamina.^61^ Additionally, recent research has indicated that as cells enter confined spaces, they become mechanically softer.^62^ As cells successfully squeeze through the dense TME, the persistent deformation of the nucleus has shown to cause nuclear envelope (NE) rupture and muta-genesis, leading to more aggressive phenotypes and an activation of invasive programs.^63,64^ These alterations may be regulated by confinement-induced heterochromatin formation and chromatin accessibility, which regulate transcriptional activity and promote pathways that support tumor invasion and DNA damage response.^65^ Confined migration has also been linked to therapy resistance in breast cancer and glioblastoma, with physical confinement leading to chemotherapy and radiotherapy resistance via a promotion of cancer stem cell phenotypes and enhanced drug efflux proteins. Within the metastatic pathway, cancer cells intravasate vasculature to travel to distant target tissues/organs. Generally, cells experience anoikis (programmed cell death via loss of cell adhesion) when they become detached, but cancer cells leverage several mechanisms to evade anoikis through upregulation of Ras/ERK and PI3K/Akt pathways, activation of TrkB signaling, and enhanced autophagy.^66–68^ Interestingly, recent work has shown that mechanical stress through confined migration was sufficient to promote resistance to anoikis.^56^ Physical confinement lowered apoptotic caspase activation through upregulated Inhibitory Apoptosis Proteins, facilitated immune cell evasion, and enhanced cell migration *in vitro* and *in vivo*.^56^ The collective impact of confinement-induced changes shows that confined migration promotes cancer cell metastasis through reduced nuclear stiffness, transcriptional changes, enhanced invasive potential, and therapy resistance.

Emerging reports have also linked the dense TME with a reduction in effective immune surveillance, which provides a clearer path for tumor invasion and metastasis. The infiltration of migratory capacity of T cells is significantly hampered by physical confinement in the TME. This phenomenon is seen in several cancers, including ovarian, lung, and pancreatic cancer, where T cells primarily accumulate in stromal regions and cannot penetrate deep within the tumor tissue.^69^ Regions with elevated ECM density and stiffness trap T cells and reduce infiltration. Although confined migration in immune cells is still an active area of investigation, several insights have been gained, showing that immune cells can modulate nuclear stiffness and deformation, reorganize the actin cytoskeleton and contractility, and alter cell adhesion dynamics to successfully infiltrate dense environments.^70,71^ Dendritic cells can generate an actin cage while breaking down the nuclear lamina to enable increased nuclear deformation and successful confined migration.^72^ Neutrophils have lower nuclear stiffness compared to other immune cells and are thus most effective at confined migration.^73^ T cells and mature dendritic cells lean on formin-based mechanisms to generate aligned actin structures and leverage myosin II-dependent contractility to squeeze through tight spaces.^74,75^ T cells have also been shown to rely on small and dynamic focal adhesions to support transit confined environments.^76^ Although many of these mechanisms are effective under some level of confinement, they begin to fail as cells encounter much smaller pores (<1 *μ*m).^77^ Thus, while confined migration generates pro-invasive changes in cancer cells, anti-tumor immune cells are significantly hindered under tight physical confinement, leading to successful cancer metastasis and spread.

Efficient confined migration supports many aspects of biology, including development and wound healing, and can assist cancer progression through cancer cell invasion and immune cell exclusion. Ongoing work is beginning to provide mechanistic insights toward the impact of persistent or high levels of confinement on nuclear integrity and the development of a variety of disorders. A recent report revealed that confined migration in normal physiological migration of neurons generated massive DNA double-stranded breaks but without detectable nuclear-envelope rupture.^78^ These double-stranded breaks were repaired in normal conditions via the non-homologous end-joining (NHEJ) pathway and led to no cell death; however, mutant mouse models (NHEJ-deficient condition) that disrupted the repair mechanism led to the eventual emergence of motor deficits, consistent with neurodegenerative disease. Additionally, recent work has identified the potential link between the endosomal sorting complex required for transport (ESCRT) pathway and nuclear envelope repair in cells that have undergone confined migration.^79^ Faulty nuclear repair of the nuclear envelope via ESCRT leads to DNA damage and altered exchange of cellular materials, which significantly impairs cell function and has been linked to neurodegenerative diseases and muscular dystrophy. Importantly, the function of these nuclear repair mechanisms is affected with age and offers clues to the etiology of neurodegenerative disease. These mechanistic studies unveil new perspectives on the importance of nuclear repair mechanisms in response to confinement. Continued investigations into confined migration mechanisms are crucial for designing strategies that can support regenerative medicine and healing, reduce cancer cell invasion, and promote anti-tumor immune cell infiltration.

## EXPERIMENTAL PLATFORMS FOR CONFINED MIGRATION

Elucidating confined migration mechanisms is challenging, but significant insights can be gained through bioengineered *in vitro* platforms and computational modeling. Cells *in vivo* exist within complex microenvironments composed of neighboring cells and ECM components that present diverse structural and mechanical cues.^1,59,80^ To study confined migration under controlled conditions, advanced 2D and 3D *in vitro* platforms are required to mimic the intricate topographies and physical constraints that influence cellular plasticity and migratory behavior. A variety of engineered *in vitro* systems, including polydimethylsiloxane (PDMS)-based platforms, microprinting strategies, and hydrogels, have been developed to recapitulate key features of the native microenvironment, enabling systematic investigation of how the biophysical properties of surrounding tissues may regulate cell migration and behavior (Fig. 2). Table I summarizes the type of confinement applied and the range of control available for each engineered platform.

### PDMS-based platforms for confined migration investigations

PDMS is an elastomer widely used to fabricate microfluidic devices for studying cell migration in controlled environments. PDMS offers several advantageous properties, including optical transparency, gas permeability that permits oxygen diffusion, and the ability to be readily molded using patterned silicon wafers. These features enable the fabrication of precisely defined architectures that mimic aspects of the physical microenvironment. Within these devices, features can be functionalized with ECM proteins to promote cell adhesion and migration or left minimally coated to simulate “barrier-free” tracks that resemble structural pathways *in vivo*, such as vascular conduits or clefts between tissue fibers. Common designs for PDMS confined migration platforms include lateral confinement and vertical confinement devices. Lateral confinement devices include microchannels, which simulate sustained confinement such as that imposed in dense ECM or aligned tissue tracks, and post-pinch-point platforms, which simulate cell extravasation/intravasation through vascular and matrix environments. Vertical confinement platforms simulate planar deformation seen in transendothelial migration or through peritoneal cavities. Importantly, the dimensions of these devices can be precisely tuned to impose defined levels of spatial confinement, allowing researchers to systematically investigate how physical constraints influence cell morphology and migratory mechanisms. Although PDMS-based devices are highly useful for confined migration studies, the tunability of mechanical properties is limited, and devices are often stiffer than normal tissue. Additionally, PDMS is non-degradable, limiting studies considering the role of proteases in modes of confined migration.

#### Lateral confinement platforms—Microchannels

A common way to investigate lateral confined migration is to impose physical constraints via microchannels, which are typically straight, shaft-like structures with varying widths ranging from 3 to 50 *μ*m [Fig. 2(a)]. Typically, the vertical height of these channels is set between 3 and 6 *μ*m, providing cross sectional areas that mimic the varying pore sizes (9–30 *μ*m^2^) in highly confined environments *in vivo*. Open faced channels have also been used, which allows for mechanical probing of cells under confinement.^62,83^ Within these platforms, experimental findings show consistently that cells exhibit reduced migration speeds with increasing levels of confinement.^83,84^ Compared with 2D environments, cells migrating within microchannels display a sliding mode of migration with minimal changes in cell length. Additionally, there is a biphasic relationship between migration speed and channel width, with cells migrating faster with increasing channel widths from 3 to 25 *μ*m but progressively decreasing in larger channel widths.^85^ Introducing complex microchannel geometries, such as constrictions or obstacles, provides a promising approach to study how cells make migratory decisions under physical confinement. In one study, breast tumor cells were introduced into a 15-*μ*m-wide channel that constricted to a 3.3-*μ*m-wide pore.^86^ Cells exhibited a delay at the constriction, with dwell times varying according to pore size, and some cells reversed direction upon encountering the barrier. When presented with a choice between a 10- and 3.3-*μ*m-wide channel at a 90° bifurcation, over 90% of cells chose the wider path; however, when the narrow channel was aligned collinearly with the original channel, only 68% of cells opted for the wider path.^87^ These observations highlight the interplay between microchannel geometry, cell signaling, and migratory decision-making. Mechanistically, cells may generate local chemical gradients by uptaking growth factors to guide migration, while some immune cells, such as neutrophils, can sense hydraulic resistance and preferentially move through paths of least resistance. Whether tumor cells can similarly use mechanosensitive hydraulic strategies remains unknown. Studies have also used microchannel devices to investigate the specific roles of lateral and vertical confinement in cell migration by varying the dimensions of lateral and vertical channel widths to control the aspect ratio of the pore. Wisniewski *et al*. discovered that confined migrating cells exhibit a top-to-bottom polarity, termed dorsoventral polarity, that activates different cytoskeletal regulatory pathways depending on the axis of confinement.^88^ Vertical confinement, which is imposed from the dorsal (top) side of the cell to the ventral (bottom) side of the cell, induces nuclear stiffening in a myosin II-dependent manner, resulting in slow bleb-based migration driven by hyperactive RhoA. Lateral confinement, however, does not produce a myosin II-dependent change in nuclear stiffness and maintains a steady RhoA activity, leading to faster mesenchymal-like migration. Through modulation of different combinations of vertical and lateral width dimensions, the authors determined that distinct migration path geometries *in vivo* regulate varied myosin II-dependent mechanisms that regulate the spatial activity of RhoA to facilitate confined migration through complex confined environments.

#### Lateral confinement platforms—Post-pinch-point devices

Lateral confinement can also be applied via post pinch-point PDMS platforms [Fig. 2(a)], which simulates the discontinuous confinement path that cells are subjected to during *in vivo* migration. These devices use posts to provide tight constriction followed by a less confined region, which can reproduce the dynamic processes that may occur during confined migration.^6,89^ Using these systems, many insights have been gained toward the understanding of the myosin-mediated translocation of the nucleus, nuclear rupture processes, and DNA repair mechanisms following confined migration. Researchers have used fluorescent reporters such as nuclear localization signal (NLS)-tagged probes to visualize nuclear envelope (NE) ruptures during pinch-point confinement.^64^ By observing the NLS signal during transit through confined posts, insights regarding nuclear deformation and NE rupture are recorded. During pinch-point confinement, an increase in intranuclear pressure causes nuclear blebbing, followed by increased cytoskeletal tension, which propels the nucleus through the posts. Under tight confinement of <6 *μ*m, over 90% of cells will experience NE rupture, which can fragment chromatin and enhance DNA double-strand breaks.^64,65^ Importantly, nuclear repair mechanisms mediated through ESCRT-III proteins restore NE integrity and seal any gaps within the NE. Within this intricate process of nuclear deformation, DNA damage, and NE repair, several lines of inquiry have emerged to dissect the impact of confinement on the etiology of neurodegenerative disease, genomic instability of metastatic cancer cells, and more recently its effect on mechanical memory.^64,79,90^

#### Vertical confinement platforms

Pillar-based strategies have also been developed to induce confinement by applying compressive forces vertically to cells [Fig. 2(a)]. Vertical compression deforms the nucleus but enables planar migration, which is reminiscent of confined surfaces *in vivo* such as those in the peritoneal layers covering organs. In this approach, a pillared PDMS array is lowered over a bed of cells, confining them to defined heights.^89,91^ These systems can restrict cells to heights as small as 3 *μ*m with approximately 100 nm precision over large areas. These platforms enable confinement to be applied across a population of cells, allowing and, importantly, being removed, allowing for analysis using standard molecular biology tools, including immunostaining, western blotting, and qPCR. Using this approach, researchers have been able to study nuclear lamina regulation of blebbing and thresholds of lamina breakage and associate these changes with gene program alterations. Additionally, strategies to increase its throughput in multi-well plates can accelerate insights toward molecular changes to cells under confinement. As a result, pillar-based vertical confinement assays provide a useful platform for investigating cellular responses to mechanical compression, including nuclear lamina rupture, alterations in gene expression pathways, and confinement-induced transitions from mesenchymal cells to a more migratory state.

#### Combinatorial approaches

PDMS-based microfluidic platforms also allow for the introduction of other molecular cues, including cytokines, chemokines, and electrical fields. For example, devices including a fetal bovine serum (FBS) gradient through 10-*μ*m-tall channels with varying widths ranging from 3 to 50 *μ*m revealed differential behavior between metastatic and non-metastatic cells, highlighting the potential of such platforms for screening metastatic potential.^92^ Additionally, microchannel devices can be adapted for pharmacological studies by introducing drug candidates into a chemotactic gradient. Zhang *et al*. presented 3 × 120 microchannel-incorporating microchambers, which were used to simultaneously test up to nine pharmacological inhibitors, demonstrating the potential of high-throughput microchannel migration assays for drug screening.^93^ Direct current electric fields, typically in the range of 0.5–5 V/cm, can guide cell migration *in vivo* and play important roles in processes such as wound healing and metastasis. Studies have investigated the combined effects of physical confinement and applied electric fields on cell migration.^94^ These studies indicate that the influence of electric fields is generally secondary to topographical cues imposed by confinement. Interestingly, exposure to an electric field has been observed to reduce overall migration speed while increasing directional persistence, suggesting that electrical cues can modulate migratory behavior without overriding the dominant effects of the physical microenvironment.

### Micropatterned lines and islands for confined migration investigations

Another approach to study confined migration involves microcontact printing and its variations, which can laterally constrain cells on a 2D substrate and impose defined shapes on stationary or migrating cells [Fig. 2(b)]. In the traditional method, an elastomeric stamp is “inked” with a protein and then pressed onto a substrate to transfer the pattern, with non-stamped regions subsequently filled with a cell-repellent polymer. More recent techniques use optical ablation to remove non-adhesive polymers, which are then backfilled with cell-adhesive proteins, enabling smaller feature sizes down to approximately 1 *μ*m in width.^95^ In addition, local stiffness or composition can be tuned by selectively cross-linking polymers in specific regions. These patterned lines can also serve as simplified models of ECM fibers, providing a controllable platform to investigate how geometry, adhesion, and substrate mechanics influence cell migration. It is important to note, however, that these platforms are limited to adhesion-based migration and do not physically constraint cells in a 3D context.

#### Micropatterned one-dimensional lines to study cell migration

One-dimensional (1D) migration can be modeled using lateral confinement, which restricts cells along a single axis. When the width of the adhesive line is narrower than the cell body, cells migrate back and forth along the line, effectively mimicking linear migration along ECM fibers. Doyle *et al*. used 1D patterned fibronectin lines with concentrations ranging from 0.5 to 1000 *μ*g/ml and showed that cells on these linear tracks move faster than cells on two-dimensional substrates, independent of ligand density.^95^ However, when line widths exceed approximately 5 *μ*m, 1D migration is lost and migration speed decreases. These assays have also provided insight into the relationship between cell shape, cytoskeletal organization, and nuclear morphology and translocation, as well as allowing quantitative measurements of migration speed and directional persistence.

#### Microcontact-printed islands

Cell polarization has been identified as a key early cue for directed migration, and the influence of confined geometry on polarization can be investigated using microcontact-printed “islands” that define sites of cell adhesion. Studies have shown that cells of identical overall shape can establish distinct polarity orientations depending on the adhesion pattern. When cells are confined to elongated shapes via microcontact printing, they tend to migrate along the long axis of the cell upon release, demonstrating that imposed geometry can dictate migration direction.^96^ Microcontact printing can be extended to other substrates, such as polyacrylamide gels or micro-post arrays, enabling studies of how confinement influences the generation and orientation of traction forces.^97,98^ Microprinting and micropatterning techniques can also be used to generate rigid islands of defined geometries through localized cross-linking on otherwise non-adhesive polyacrylamide hydrogels. These micropatterned stiffness islands provide a platform to investigate how cells sense and respond to spatial variations in substrate rigidity. Studies have shown that fibroblasts are able to bridge gaps between adhesive regions on stiff substrates but not on soft ones, demonstrating that cell spreading and migration are strongly influenced by the mechanical properties of the underlying matrix.^99^ Confinement on these patterned substrates also reduces focal adhesion size, consistent with observations in narrow microchannels and on one-dimensional patterned lines. Mechanistically, rigidity sensing and cell spreading depend on cytoskeletal regulators, including Rac1, Arp2/3, and myosin II-mediated contractility. Inhibition of myosin II disrupts rigidity sensing, allowing cells to spread across otherwise nonpermissive soft substrates.^99^

### Tunable hydrogels for confined migration investigations

Recently, studies have been able to expand confinement assays by incorporating mechanically tunable hydrogels to independently examine the effects of microchannel dimensions, substrate stiffness, and ligand density [Fig. 2(c)]. These systems complement traditional 3D migration assays by providing defined migration tracks while maintaining more physiologically relevant stiffness compared to PDMS microchannels. Although fabrication is more complex, techniques such as optical patterning and micro-molding enable the creation of these hydrogel-based confinement platforms.^81,82^ There are still limitations to current techniques, as some parameters, such as hydrogel stiffness and porosity, are often coupled, preventing the independent investigation of each parameter. Thus, continued investigations into hydrogel fabrication approaches are needed.

#### Variable pore sizes via controlled polymerization

To better model the structural variability of fibrillar ECM, which can range from loosely organized to densely packed collagen networks, several approaches have been developed to control pore size and fiber alignment in collagen gels. These include adjusting polymerization conditions or using collagen with varying telopeptide content to modify fibril assembly.^100,101^ However, a recognized limitation of these systems is that the pore sizes achievable in 3D collagen gels are often smaller than those found in native tissues *in vivo*.^101^

#### Photopatterned gels

Photopatterning is a technique that uses light to selectively modify or remove regions of hydrogels with high spatial and temporal precision, enabling patterning with around 1 *μ*m lateral and 3–5 *μ*m vertical resolution. This allows for control of ECM proteins and growth factors within 3D matrices to guide cell migration or enables localized degradation to create features such as microwells, grooves, or 3D migration tracks. These tracks can be used to study tumor cell invasion and cell migration through confined spaces, including migration that occurs independently of matrix metalloproteinase (MMP) activity.^81^ Furthermore, because photopatterning enables independent control of ligand presentation and microtrack geometry, it provides a powerful platform for investigating how cells respond to distinct biochemical cues and for studying specific cell populations within 3D gels.

#### Micro-molded collagen and polyacrylamide hydrogels

Microfabrication techniques can also use polymers other than PDMS to mold structures from patterned masters, enabling independent control of substrate stiffness and microchannel geometry in both degradable and non-degradable materials. For example, microtracks formed in collagen matrices increase the migration speed of epithelial and cancer cells compared with unpatterned collagen gels, although migration speed within the tracks is largely independent of collagen concentration.^102^ Similar approaches using molded polyacrylamide troughs that confine cells on three sides have shown that cell migration depends on both channel width and substrate stiffness, with migration speed increasing with stiffness in narrow channels and displaying a biphasic response to stiffness in wider channels.^103^

### Emerging techniques and future platforms for confined migration investigations

The majority of confined migration investigations have employed platforms that test different degrees of confinement by changing the physical dimensions of the systems. This includes adjusting channel dimensions for microchannels, setting static dimensions for pore size, and tuning hydrogel systems by adjusting formulation and cross-linking strategies. However, these systems do not appropriately include the dynamic nature of the ECM *in vivo* and the time-dependent effects of migration. Several recent reports have highlighted the importance of substrate rigidity dynamics and the effect of matrix viscoelasticity on cell migration. Yang *et al*. identified the innate capacity of cells to migrate through varying mechanical environments, where substrate stiffness may fluctuate.^104^ Using dynamic and reversible photo-responsive hydrogels, they fluctuated mechanical stiffness from 1.6 to 2.2 kPa in 1-min cycles and found that cells migrated more quickly when compared to cells cultured statically at 2.2 kPa. This enhanced migration potential was regulated by the balance of forces between the inner and outer forces of the cell. Importantly, these findings were validated and can be predicted through a quantitative computational model. Matrix viscoelasticity has also been examined in the context of migration, revealing that stress relaxation regulates actomyosin-driven remodeling and migration. In a recent report, slow stress relaxation collagen gels impair dendritic cell migration and activation.^105^ Furthermore, confinement within this viscoelastic context induced mechanisms tied with cell memory, including epigenetic changes such as chromatin accessibility. Matrix plasticity, the dynamic, malleable characteristics of matrix where cells can deform and alter matrix ultrastructure and porosity, has also been integrated within hydrogel systems to examine its effect on cell migration. Using an interpenetrating network with controlled plasticity, Wisdom *et al*. identified a novel mode of protease-independent migration, where cells extend invadopodia protrusions to physically open up micrometer-sized pores and channels to migrate in confined microenvironments.^106^ Collectively, these advances in hydrogel systems reveal that several dynamic mechanical inputs play important roles in confined environments, necessitating further investigations into confined migration within these dynamic systems. To gain stronger control over confinement parameters in dynamic hydrogel systems, future work should integrate micro-molding techniques using microfabricated silicon masters to generate topographical patterns such as microchannels to mold these dynamic hydrogel designs onto.^103^ This combinatorial approach will assist in the next wave of confined migration studies to illuminate the impact of dynamic mechanical inputs on confined migration.

## MATHEMATICAL MODELING OF CONFINED CELLULAR MIGRATION

Because multiple aspects of cellular migration under confinement are largely investigated at the single-cell level, it is difficult to examine confined migration in larger-scale settings. Mathematical modeling may help clarify how confinement influences tissue-scale behaviors. Mathematical modeling and computational simulation are especially suited for complex system analysis and have been extensively applied to study biological phenomena over the past several decades. Specifically, modeling and simulation provide a framework for translating experimentally observed biological processes into quantitative relationships. Mathematical modeling has described multiple phenomena involved in confined cellular migration, including adhesion strength, actin polymerization rates, myosin contractility, nuclear stiffness, channel geometry, and matrix properties.^107–111^ Each mathematical modeling approach, once calibrated to corresponding *in vitro* measurements, predicts how variations in specific cellular properties affect confined cellular migration by systematically varying individual parameters that may be difficult to isolate experimentally. Validating these predictions with experimental setups would help confirm model accuracy and generalizability. Consequently, mathematical modeling is useful when iterative cycles of prediction, experimentation, and validation refine a system’s mechanistic understanding.

Mathematical models describing confined cellular migration operate across multiple organizational scales, ranging from molecular processes to whole-cell migration dynamics to tissue-level behaviors that emerge from cell–cell and cell–environmental interactions. At the molecular scale, models focus on biochemical and mechanical processes associated with the cytoskeleton.^112,113^ The cellular scale utilizes molecular phenomena and examines whole-cell migration dynamics and the integration of molecular machinery to explain how confinement alters cell behavior.^60,114^ Confined cellular migration at the cellular scale can be used to describe collective migration between cells and cell–cell interactions and how they further contribute to behaviors at the tissue scale^21,115^ that span thousands of cells. Table II summarizes the mathematical models surveyed in this review and their biological relevance to confined cellular migration.

### Molecular-scale modeling

Molecular-scale models capture cytoskeletal force transmission and mechanochemical signaling essential for confined migration. The Molecular Motor Clutch Model (Molecular MCM), a well-established representation of cells using a “clutch-like” action to migrate,^126^ focuses on cellular mechanics and physiology to explain migration in confined environments. In Prahl *et al*.,^107^ glioma cells were modeled to use a motor-clutch force-transmission mechanism to migrate within confined spaces, and the model predicted migration velocities consistent with *in vitro* observations. The study proposes that glioma confined cellular migration involves dynamic cytoskeletal self-assembly in the absence of cell adhesion molecules and suggests that a physically confined environment is crucial for efficient cell movement. Motor-clutch force transmission has also quantified the effect of altered integrin adhesion phenotypes on the invasiveness of confined metastatic breast tumors, as shown in Mekhdjian *et al*.^108^ These results demonstrate that Molecular MCM can be adapted for multiple cell types and experimentally fit confined cellular migration data.

Cancer-linked biomarkers can be targeted therapeutically to modulate cancer cell migration, which often occurs in confined environments. In an *in vitro* experiment guided by image-based simulations in Rosen *et al*.,^127^ glial cells performing confined cellular migration were disrupted by microtubule-targeting agents, and, similarly, Molecular MCM has been used to predict therapeutic responses *in vitro*.^116,126^ Furthermore, confined migration is linked to regular changes in RhoA guanine nucleotide exchange factor (GEF-H1),^117^ which activates RhoA proteins to regulate actomyosin tension in the molecular clutch used during cellular movement.^126^ In Lee *et al*.,^117^ GEF-H1’s oscillations were predicted and fitted to a system of ordinary differential equations (ODEs) describing Ca^2+^-mediated GEF-H1 expression. Predicting and fitting this biomarker’s oscillations further demonstrates the model’s ability to couple closely with biological processes compared with models such as the Cellular Potts Model (CPM).

Although insightful for cellular movement and migration, the Molecular MCM can easily generate complex sets of differential equations as it is scaled from molecular to cellular implementations.^126^ Even so, Bangasser and Odde^120^ derived a single ODE to address computational complexity and to expand simulation and model organizational complexity for multi-scale integration. Since 2013, this ODE approach has enabled more sophisticated models to incorporate additional biological phenomena into the Molecular MCM framework, such as the multi-scale model presented in Venturini and Saez.^118^ The model extends the classic motor-clutch framework to include adhesion-chain mechanics and mesoscale (nm to *μ*m) adhesion-complex behavior, bridging the molecular and cellular scales in a computationally efficient manner. However, exponential increases in computational complexity prevent simulations of single adhesion complexes from scaling to cell-wide scales.

### Cellular-scale modeling

Cellular-scale models integrate molecular machinery to predict whole-cell migration dynamics under confinement, capturing how geometric constraints shape cell morphology, how cytoplasmic and nuclear deformability modulate migration efficiency, and how both intracellular and extracellular mechanics influence locomotion strategies.

#### Cellular Potts Model

The Cellular Potts Model (CPM) provides a discrete, lattice-based framework for simulating cell shape changes and migration under confinement, enabling analysis of nuclear mechanics and motility. It is a commonly used cellular- and tissue-scale model that represents cells in a time-discretized grid,^128^ describing cellular migration based on kinetic favorability (as encoded by the Hamiltonian operator).^109,129^

The use of a CPM in Li and Lowengrub^119^ demonstrated how kinetic factors influence cellular behavior under confinement. Their simulations showed that increased cell motility leads to smaller cells, and increasing cellular compression tends to reduce growth rates.

As the nucleus is the largest and stiffest organelle,^9^ it is the primary physical barrier limiting confined cellular migration; therefore, extrinsic factors such as confinement geometry and the extent of confinement are key parameters. Additionally, to better capture the role of nuclear mechanics in confined cellular migration, Kumar *et al*.^109^ modeled single cells in a CPM by representing the nucleus and cytoplasm as distinct components. This approach enabled analysis of how the deformability of both components influences migration under confinement. Using stochastic Monte Carlo simulations, the study found that cellular deformability had a greater impact on migration efficiency than nuclear deformability, highlighting the importance of whole-cell mechanics in navigating confined environments.

CPM also offers a highly configurable framework for investigating confined cellular migration, allowing variation of mechanical and biochemical parameters without requiring significant changes to the underlying model structure. CPM simulations have been used to investigate how cytoskeletal stiffness may influence confined cellular migration. For example, the CPM by Giordano in Ref. 120 suggested that increasing muscle-fiber elasticity could slow migration through dense ECM. Such simulations provide a way to hypothesize how an environment’s biomechanical properties might constrain confined cellular migration, even when direct experimental validation is challenging. This capability makes CPM particularly useful for probing spatially heterogeneous phenomena and testing how environmental or cell structural properties influence migration dynamics.

To determine how specific biophysical determinants of confined cellular migration (e.g., adhesion, volume conservation, environmental stiffness, etc.) affect migration, CPM expresses these parameters as additive energy terms within a single Hamiltonian function and updates cell configurations using a Metropolis algorithm. This modularity simplifies configurability when exploring different conditions, while other models, such as continuum frameworks, often require reformulating and solving coupled nonlinear partial differential equations (PDEs) when constitutive relationships and interactions change. Thus, CPM enables researchers to investigate confined cellular migration under a wider range of conditions than other approaches.

While CPM is effective for simulating migration dynamics and cell shape changes, key limitations remain. CPM parameters often lack direct physical units, making biological interpretation and calibration against experimental data potentially difficult.^129^ Furthermore, current CPM accuracy in mimicking complex biological phenomena (e.g., confined cellular migration) remains relatively unexplored, with limited parameter sets that may neglect more complicated or long-term behaviors.^128^ These constraints suggest that, beyond calibration, capturing confined cellular migration dynamics may require strategically increasing model complexity or integrating CPM with multi-scale frameworks to incorporate mechanical and biochemical factors.

#### Continuum models

Continuum modeling, in contrast to CPM, mathematically represents a continuous physical substance rather than discrete particles.^115^ Model-governing PDEs describe the cell’s physical properties and how they change over time. Like the Molecular MCM, continuum modeling is unique in its implementation of different forces and its understanding of how they relate to cell morphology and migration. Continuum models rely on phenomenological assumptions to describe constitutive relationships, such as the use of Active Gel Theory by W€ossner *et al*. to model actin networks as continuous, viscous material for cell locomotion.^121^

Aubry *et al*.^15^ used continuum modeling to classify cellular migratory phenotypes as permeative, invasive, or penetrating based on the distance covered during confined migration. The model linked these phenotypes to cellular morphology and active forces. Using these classifications, the conditions under which cells are invasive or permeative can be discerned, enabling the prediction of confined cellular migration behavior based on cellular shape and force interactions. Additionally, cellular and nuclear deformation were described using a 2D continuum model by Allena in Ref. 122, where the cytoplasm and nucleus are modeled separately as viscoelastic cellular components. Model results suggested that a cell could adjust its movement and shape to the complex geometry of the environment and migrate through confined spaces in response to mechanical interactions alone,^122^ thus potentially being a major factor in driving confined cellular migration.

Compression of the nuclear membrane is a rate-limiting step in confined migration and has been challenging to model both experimentally and mechanistically.^60,109,130^ Using continuum modeling, studies can effectively investigate the forces required for nuclear deformation and the mechanisms that prevent rupture. Estabrook *et al*.^110^ modeled the nucleus of a cell during confined migration as a continuous elastic solid,^110^ finding that the nucleus undergoes active compression by surrounding actin molecules as it enters a confined area during actin polymerization. Nuclear deformability is regulated by interconnected contributions from the nuclear lamina, chromatin organization, and linker of nucleoskeleton and cytoskeleton (LINC) complexes, all of which influence how forces are transmitted from the cytoskeleton to the nucleus during confined migration.^9,61,63,64,109^ While many nuclear deformations are reversible, severe or repeated confinement can lead to nuclear-envelope rupture, DNA damage, and genomic instability.

The *in vitro* model used by Halfmann *et al*. in Ref. 131 suggested that barrier-to-autointegration factor (BAF) may assist in nuclear envelope repair during confined migration. The study notes that failure in BAF’s biochemical role would lead to persistent damage and may promote the migration of invasive cancer cells by exposing DNA to cytoplasmic exonucleases. Including factors such as BAF in mechanistic modeling efforts would enable better prediction of how cells are compressed and released, providing a deeper understanding of the interplay of forces that allow nuclear membrane flexibility in confined environments and cancer invasiveness.

### Multicellular models

Collective migration is a complex behavior that involves processes at the cellular scale. Many mechanistic modeling techniques adapt single-cell models, such as CPM and continuum models, to multicellular scenarios because these interactions are fundamentally cellular-scale and drive tissue-level behaviors. These adaptations aim to accurately capture collective phenomena related to confined cellular migration, including leader–follower dynamics,^132,133^ polarity-driven coordination,^19^ and ECM remodeling.^125^ Such mechanisms are crucial for essential biological processes like embryogenesis,^80,134,135^ wound healing,^135,136^ and cancer invasion.^135,137^

Collective migration relies on coordinated contractile processes and exhibits front-to-back polarity, with leading cells at the front edge.^138^ Leader–follower behavior between cells has been modeled using a spring-based phenomenological model of the cellular membrane in Peng *et al*.^139^ The model suggested that cells pushing on a leader cell during collective migration increase its migration speed, implying that collective migration could increase invasiveness.^139^ Giordano used a CPM in Ref. 120 to demonstrate collective and coordinated migration in a confined context, where leader–follower behavior between two cells was observed. The two cells exhibit malignant tumor characteristics, and the leader cell used matrix metalloproteinases (MMPs) to guide both cells through the ECM barriers between muscle fibers. Experimental studies have reinforced these insights. Jain *et al*.^132^ showed that epithelial cells confined to ring-shaped tracks exhibit Rac1-dependent front-rear polarity, which propagates to neighboring cells and drives coordinated circular motion. Using a combination of experiments and a minimalistic particle-based model, this study suggests that the polarization of a single cell to move could be a key mechanism in driving collective migration in confinement.

Furthermore, it has been demonstrated *in vitro* that cells can interconvert between single- and collective-migration modes, depending on whether migratory channels are spaced closer together (single) or farther apart (collective).^19,99^ Additionally, Szabo *et al*.^34^ developed a CPM-based model of neural crest cells (NCCs)^34^ to study collective migration under confinement. The model predicted the optimal confinement width for a given cluster size, demonstrating the importance of geometrical confinement for efficient collective cell migration *in vivo*. While this model predicts optimal confinement width for clusters, its assumptions are specific to confined NCC streams and do not generalize to larger, unconstrained tissues. Furthermore, Bruckner *et al*.^124^ determined, through *in vitro* and mechanistic modeling, that intercellular interactions during confined cellular migration differ between non-cancerous (repulsion and friction) and cancerous (attraction and anti-friction) cells, as modeled using a stochastic equation of motion. Therefore, a general mathematical framework for confined cellular migration must account for how cells interact in both modes, as the underlying force dynamics and mechanical interactions differ between them.

Pragmatically, collective cellular migration is involved in many biological phenomena, such as wound healing and embryogenesis. Zhao *et al*.^125^ described wound healing^125^ within a viscoelastic model that contextualizes the cell–substrate mechanics involved in both intracellular and extracellular components. These mechanisms focus on cellular clusters and behaviors, including leader–follower behavior, Rac signaling, and mechanochemical feedback.^125^ Similarly, Comlekoglu *et al*.^123^ use a CPM to study collective migration of ~64 *Xenopus* mesendoderm (embryonic frog) cells, identifying that cohesotaxis, tissue geometry, and cell intercalation are key parameters in collective cell migration during embryogenesis, a confined process.

## MATHEMATICAL MODELING OF CONFINED CELLULAR MIGRATION EFFECTS AT THE TISSUE SCALE—CHALLENGES AND APPROACHES

While mathematical models have been developed for both cellular and tissue-scale migration, the role of confined cellular migration and multiscale interactions in tissue-scale models remains under-explored, despite their potential to demonstrate emergent cellular behaviors. A focus on tissue-scale mechanics is essential because it represents the clinically relevant scale at which therapeutic efficacy and emergent confined cellular migration phenomena are observed and measured, such as in assessing tumor cell metastasis in patients.^135,137^ These gaps underscore the need for tissue-scale modeling of confined cellular migration, multi-scale integration of molecular-, cellular-, and tissue-scale processes, and machine learning approaches to improve model qualification and accuracy.

### Tissue-scale modeling efforts

While confined cellular migration plays a critical role in many biological processes, it has not yet been fully represented in tissue-scale models (to the authors’ knowledge). CPM-based models can capture tissue deformation and collective migration,^35,140^ but confinement is typically simplified or omitted at larger scales.^109^ Increasing the number of interactions among differential equations hinders Molecular MCM and continuum approaches from describing tissue-scale phenomena, resulting in steep computational costs when extended to tissue-level domains, often requiring simplifications that eliminate confinement-specific mechanics.^115,126^ These limitations reflect a broader challenge: incorporating spatial heterogeneity and mechanical constraints into large-scale simulations while maintaining computational feasibility. As a result, tissue-scale modeling efforts provide valuable insight into cellular locomotion but may fall short of incorporating molecular- and cellular-scale confined cellular migration effects that could be crucial to accurately represent larger-scale phenomena.

### Multi-scale models and hybrid approaches

To address the current limitations of tissue-scale models, multi-scale and hybrid models could integrate complex relationships in confined cellular migration that are often omitted in tissue-scale modeling efforts. Both approaches can combine cell-based mechanics, tissue-level forces, and biochemical signaling to capture emergent behaviors under confinement.

Recently, a multi-scale model was designed by Shu and Kaplan^141^ to couple cell-intrinsic mechanics, intracellular signaling, and the surrounding environment to describe remodeling and collective migration under confinement. Specifically, the model integrates focal adhesion and signaling dynamics (molecular scale), cytoskeletal traction and nuclear deformation (cellular scale), and environmental properties such as viscoelasticity, channel geometry, and wall friction to produce emergent behaviors. The framework demonstrated how force transmission through the cytoskeleton and nucleus under confinement can generate emergent behavior at a cellular scale by incorporating molecular mechanics, underscoring the need to simulate confined cellular migration processes across multiple scales within models.

To better achieve multi-scale modeling, hybrid approaches can bridge discrete and continuous models. For example, Hirway *et al*.^142,143^ defined a hybrid CPM to model tissue formation during epithelial–mesenchymal transition (EMT) using a multi-component model. This model combines CPM for cell shape and motility, a finite element model for substrate mechanics, a first-moment-of-area construct for traction forces, and ODE/PDEs for intracellular EMT signaling and extracellular cues.^142,143^ Similarly, Keijzer *et al*.^111^ designed another hybrid model^111^ to interpret cell spreading in response to ECM fibril stiffness, where CPM captured dynamic cell shape changes, molecular dynamics simulated ECM fibers, and ODEs modeled focal adhesion turnover. However, hybridizing models may increase calibration difficulty and computational complexity. Calibrating additional model parameters arising from hybridization or multi-scale representations could require *in vivo* and *in vitro* experimentation to quantify reasonable parameter ranges and ensure model accuracy. Improved numerical analysis strategies, such as machine learning, combined with advances in parallelization, could enable simulations of millions of cells in realistic tissue geometries, making hybrid confined cellular migration models feasible at the tissue scale.

### Machine learning approaches

Machine learning approaches may further support confined cellular migration studies through trajectory classification, migration-mode recognition, image segmentation, cell tracking, parameter inference, and prediction of future cellular states from imaging data.^144–146^ For example, supervised learning approaches may improve classification of migratory phenotypes such as permeative, invasive, or penetrating, as described by Aubry *et al*.^15^ Machine learning may also facilitate integration of multimodal datasets, including imaging, force measurement, and transcriptomic data, to identify relationships that are difficult to resolve experimentally.^145^

Machine learning techniques, such as data extrapolation and predictive machine learning, could complement mathematical modeling of confined cellular migration. For example, though not yet in confined contexts, Camacho-Gomez *et al*.^144^ extrapolated two-dimensional experimental trajectories to three-dimensional migration behavior and validated predictions using light-sheet microscopy. This approach demonstrates a potential strategy to decrease the resource and time barriers involved in tissue-scale modeling.^144^ Similarly, machine learning approaches have been applied to infer potential links between measurable quantities and biological phenomena from data. In Camacho-Gomez *et al*.,^145^ machine learning methods were used to accurately predict barotactic behavior of cells, demonstrating how computational modeling can infer cell–environment interaction rules directly from experimental data. Comparable strategies could identify governing mechanisms of confined cellular migration across molecular, cellular, and tissue scales.^15,144–146^

Furthermore, machine learning could help mitigate the computational complexity of mathematical models. Using integration techniques discussed in Gherman *et al*.,^146^ a Molecular MCM, or continuum model, could potentially leverage machine learning to mitigate the higher computational burden inherent to tissue-scale modeling applications. Long-term, integration of machine learning with confined cellular migration models may facilitate exploration of novel therapeutic strategies for clinically relevant applications.^146^ Despite these opportunities with machine learning approaches, several limitations exist. Confined migration datasets are often small and generated under platform-specific conditions, creating risks of overfitting, batch effects, and limited generalizability. Additionally, machine learning models may achieve high predictive accuracy while offering limited mechanistic interpretability. Consequently, external validation using independent datasets and integration with mechanistic models will be essential for robust application of machine learning to confined cellular migration research.

## CONCLUSION

The continued advancement of experimental approaches for confined migration, such as bioengineered *in vitro* platforms and mathematical models, is expected to aid in the understanding of the mediators of confined migration in physiology and disease. These insights could generate new targets that restrict confined migration (as in cancer metastasis) or promote it (as in immune infiltration in cancer, wound healing, and regenerative medicine/cell-based therapies). Advances in drug delivery methodologies will be needed to generate targeted responses to cells of interest to minimize any nonspecific effects. Challenges remain, as current *in vitro* systems carry inherent limitations, and mathematical approaches have yet to fully integrate the biomolecular and biophysical cues within the complex tumor microenvironment at tissue scale.

As the field advances, a multi-pronged approach to combining experimental and computation insights is needed at multiple scales. Experimentally, significant insights have been gained at the cell scale, identifying the roles of various cytoskeletal elements in adhesive and non-adhesive states and the adaptability of cells to transition between mesenchymal and amoeboidal modes of migration. Importantly, computationally generated mechanistic models have confirmed many of these mechanisms. However, the next stage of development requires experimental approaches that extend into the multicellular stage with controlled techniques in 3D as well as improved *in vivo* imaging. Computationally, future efforts should focus on improving multiscale and hybrid modeling frameworks that link organizational-scale processes while remaining computationally feasible.^111,141–143^ Continued development of machine learning approaches could potentially decrease or overcome computational expense, accelerate parameter exploration, and enable more complex simulations of confined cellular migration.^144–146^ Importantly, computational approaches can inform experimentation by generating testable hypotheses, identifying governing physical mechanisms, and guiding experimental design.^107,117,141^ As modeling approaches continue to evolve, prospective validation using independent datasets and closer integration with experimental measurements will be essential to improving model robustness and predictive capabilities to elucidate mechanisms of confined cellular migration in a variety of clinical applications such as wound healing and cancer treatment.

## Figures and Tables

**FIG. 1.**
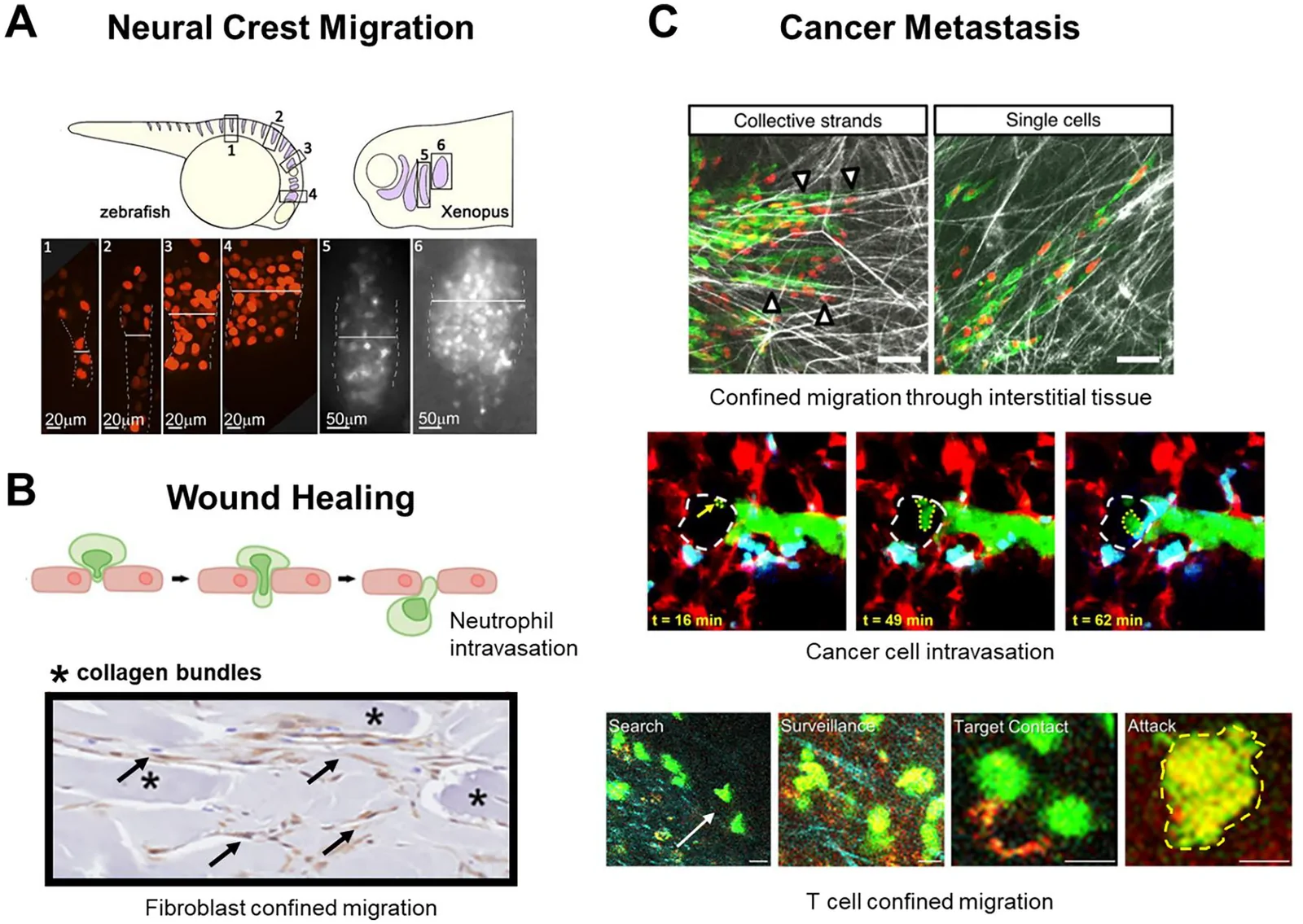
Confined migration facilitates cell invasion in development and disease. Confined migration is observed in neural crest migration through the collective swarming of neural crest cells through tracks of varying sizes of confinement^35^ (a). In wound healing, neutrophils arrive at the injury site through confined squeezing through the endothelium.^36^ Furthermore, fibroblasts that participate in scar formation will experience confinement (indicated by the arrows), especially in regions of elevated density and stiffness (b).^37^ Cancer metastasis requires invasion through the tissue interstitium^38^ to reach the tumor vasculature and intravasation and extravasation in and out of blood vessels, respectively.^39^ Anti-tumor immune cells, such as T cells, experience dramatic confinement (yellow-dotted lines) to navigate the dense tumor microenvironments and identify and attack cancer cells^40^ (c). Figures were modified and adapted with permission as indicated by citations. Reproduced with permission from Salvermoser *et al*., Blood **131**(17), 1887–1898 (2018). Copyright 2018 American Society of Hematology [panel (b)]. Reproduced with permission from Ilina *et al*., Nat. Cell Biol. **22**(9), 1103–1115 (2020). Copyright 2020 Author(s), under exclusive license to Springer Nature Limited [panel (c)]. Reproduced with permission from Entenberg *et al*., Nat. Methods **15**(1), 73–80 (2018). Copyright 2017 Springer Nature America, Inc. [panel (c)].

**FIG. 2.**
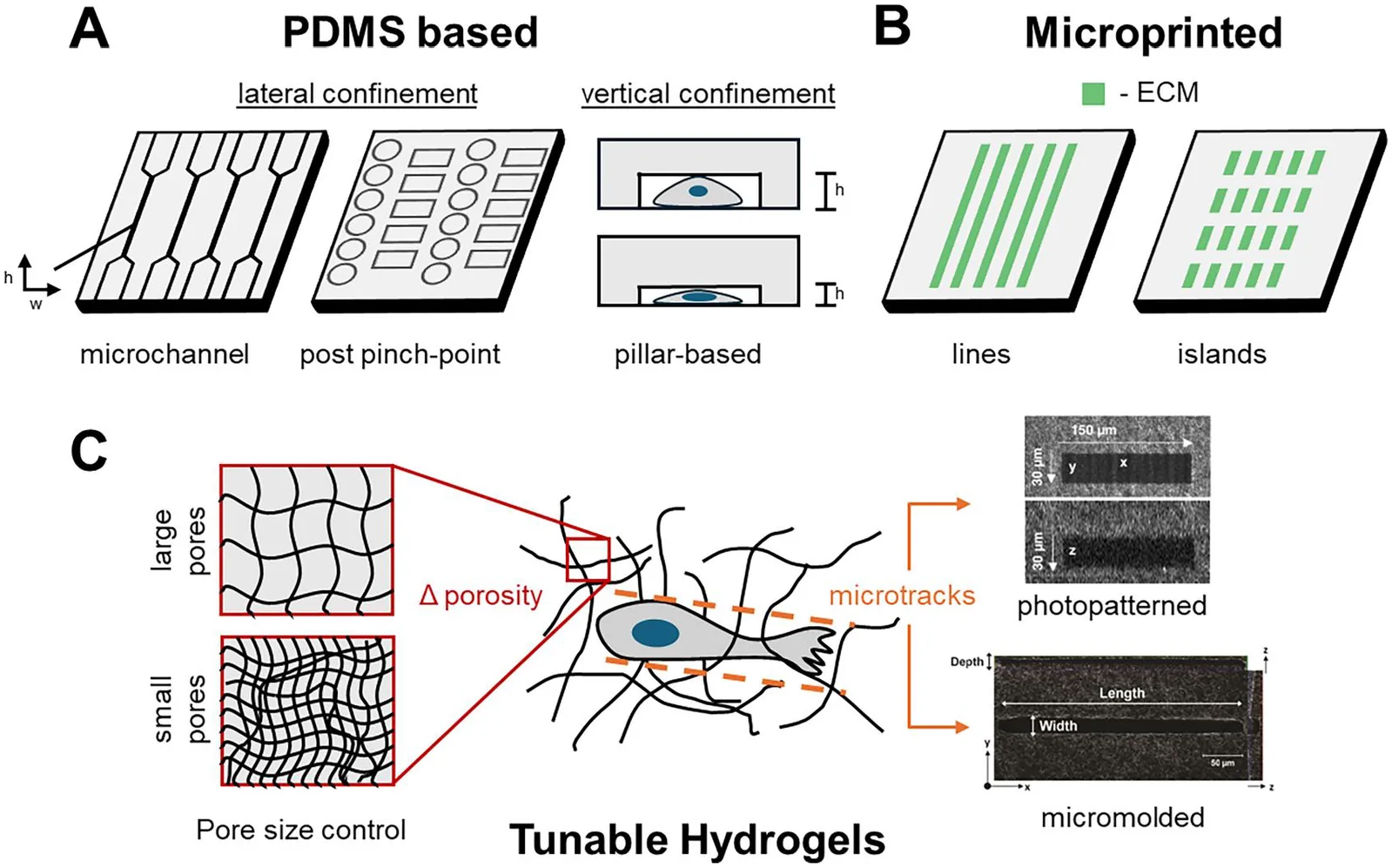
Engineering platforms for confined migration investigations. PDMS-based platforms provide lateral confinement using microchannels and posts to investigate sustained confined migration and squeezing, respectively. Microchannel platforms have dimensional control of both channel width and height, while pinch-point platforms can vary post position to adjust lateral confinement and control device height as well. Additionally, PDMS-based platforms can specifically regulate vertical confinement without lateral confinement through pillar-based designs (a). Microprinting strategies investigate confined migration through microcontact printing of ECM lines and islands over different substrates (b). Hydrogels can evaluate 3D confined migration and can be tuned to control porosity and engineered to generate microtracks through photopatterning^81^ or micro-molding approaches^82^ (c). Figures were modified and adapted with permission as indicated by citations. Reproduced with permission from Ilina *et al*., Phys. Biol. **8**(1), 015010 (2011). Copyright 2011 IOP Publishing [panel (c)]. Reproduced with permission from Kraning-Rush *et al*., Integr. Biol. **5**(3), 606–616 (2013). Copyright 2013 The Royal Society of Chemistry [panel (c)].

**TABLE I.** Summary of the types of confinement imposed using different platforms and the parameters of control.

Confinement	Platform	Parameter control	Force-measurement compatibility	Migration type Observed	Limitations
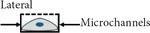	Microfluidics	Channel width Channel length Substrate stiffness Pore size	AFM, if open faced	Mesenchymal, Amoeboidal: bleb-based, pseudopodial, lobopodia, friction-driven	Rigid walls and non-degradable
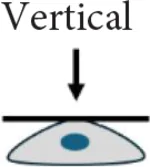	Microfluidics	Channel height Substrate stiffness Ligand density	TFM	Mesenchymal, Amoeboidal: bleb-based, pseudopodial, lobopodial, friction-driven	Lacks full 3D confinement
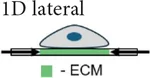	Microprinting	Line width Island size Substrate stiffness Ligand density	TFM AFM	Mesenchymal	Lacks full 3D confinement Adhesion-based migration only
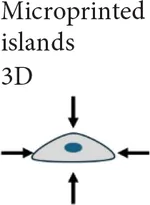	Microfluidics Hydrogels	Porosity Stiffness Viscoelasticity Degradability	TFM	Mesenchymal, Amoeboidal: bleb-based, pseudopodial, lobopodial, friction-driven	Hydrogel parameters are coupled

**TABLE II.** Summary of mathematical modeling approaches describing cellular migration phenomena.

Organizational scale	Confined migration phenomenon	Model	Biological relevance
Molecular	Motor-clutch force transmission	Molecular Motor Clutch	Predicting migration velocities^107^
			Therapeutic targeting and mechanochemical signaling^107,116,117^
	Altered integrin adhesion phenotypes	Molecular Motor Clutch	Invasiveness of metastatic breast tumors^108^
	Adhesion-chain and adhesion-complex behavior	Molecular Motor Clutch	Multiscale integration of adhesion mechanics^118^
Cellular	Single-cell mechanics	Cellular Potts Model	Nuclear vs cytoplasmic deformability^109,119^
			Migration speed under confinement^120^
		Continuum model	Cell dynamics and morphology under confinement^15,121,122^
	Nuclear deformation	Cellular Potts Model	Rupture and repair mechanisms^109^
		Continuum model	Modeling forces required for nuclear compression^110^
	Collective migration	Cellular Potts Model	Leader-follower dynamics^34,120,123^
			ECM remodeling via MMPs^120^
	Cell–cell interaction dynamics	Stochastic equation of motion	Differentiate cancerous vs non-cancerous interactive forces^124^
	Wound healing at a cellular scale	Continuum model	Modeling mechanochemical feedback^125^
	Embryogenesis at a cellular scale	Cellular Potts Model	Cohesotaxis and tissue geometry in embryonic development^123^

## Data Availability

Data sharing is not applicable to this article as no new data were created or analyzed in this study.
